# Antinociceptive effect of ethanolic extract of *Selaginella convoluta* in mice

**DOI:** 10.1186/1472-6882-12-187

**Published:** 2012-10-19

**Authors:** Pedro Guilherme S de Sá, Xirley Pereira Nunes, Julianeli Tolentino de Lima, José Alves de Siqueira Filho, André Paviotti Fontana, Jullyana de Souza Siqueira, Lucindo José Quintans-Júnior, Patrícia Kauanna Fonseca Damasceno, Carla Rodrigues Cardoso Branco, Alexsandro Branco, Jackson Roberto Guedes da Silva Almeida

**Affiliations:** 1 Núcleo de Estudos e Pesquisas de Plantas Medicinais, Universidade Federal do Vale do São Francisco, Petrolina, Pernambuco, 56.304-205, Brazil; 2 Centro de Referência para Recuperação de Áreas Degradadas da Caatinga, Petrolina, Pernambuco, 56.300-000, Brazil; 3 Departamento de Fisiologia, Universidade Federal de Sergipe (DFS/UFS), Campus Universitário “Prof. Aloísio de Campos”, São Cristóvão, Sergipe, 49.100-000, Brazil; 4 Laboratório de Fitoquímica, Departamento de Saúde, Universidade Estadual de Feira de Santana, Feira de Santana, Bahia, 44.036-900, Brazil

**Keywords:** *Selaginella convoluta*, Selaginellaceae, Analgesic, Pain

## Abstract

**Background:**

*Selaginella convoluta* (Arn.) Spring (Selaginellaceae), commonly known as “jericó”, is a medicinal plant found in northeastern Brazil. *S. convoluta* is used in folk medicine as an antidepressant, aphrodisiac, diuretic, analgesic, anti-inflammatory and it is used to combat amenorrhea, coughing and bleeding. This study was performed to evaluate the antinociceptive effects of ethanolic extract from *S. convoluta* in mice exposed to chemical and thermal models of nociception.

**Methods:**

Preliminary phytochemical analysis of the ethanolic extract was performed. The ethanolic extract from *Selaginella convoluta* (Sc-EtOH) was examined for its intraperitoneal (i.p.) antinociceptive activity at the doses of 100, 200 and 400 mg/kg body weight. Acetic acid-induced writhing, formalin injection and hot plate tests were used to evaluate the antinociceptive activity of Sc-EtOH extract. The rota-rod test was used to evaluate motor coordination.

**Results:**

A preliminary analysis of Sc-EtOH revealed that it contained phenols, steroids, terpenoids and flavonoids. In the acetic acid-induced writhing test, mice treated with Sc-EtOH (100, 200 and 400 mg/kg, i.p.) exhibited reduced writhing (58.46, 75.63 and 82.23%, respectively). Secondly, Sc-EtOH treatment (100, 200 and 400 mg/kg, i.p.) decreased the paw licking time in mice during the first phase of the formalin test (by 44.90, 33.33 and 34.16%, respectively), as well as during the second phase of the test (by 86.44, 56.20 and 94.95%, respectively). Additionally, Sc-EtOH treatment at doses of 200 and 400 mg/kg increased the latency time in the hot plate test after 60 and 90 minutes, respectively. In addition, Sc-EtOH did not impair motor coordination.

**Conclusion:**

Overall, these results indicate that Sc-EtOH is effective as an analgesic agent in various pain models. The activity of Sc-EtOH is most likely mediated via the inhibition of peripheral mediators and central inhibitory mechanisms. This study supports previous claims of traditional uses for *S. convoluta*.

## Background

Selaginellaceae Willk. is a distinctive family that includes the genus *Selaginella,* which is found worldwide and comprises approximately 700 [1] to 750 species [2]. Approximately 270 species of *Selaginella* are found in America, and the genus is widely distributed throughout America, Africa and Europe. In America, *Selaginella* can be found from northern Alaska east to Greenland, and south as far as Mendoza and Buenos Aires in Argentina. *Selaginella* are best represented in the Amazon basin, with 31 species known to grow in that region [1]. Members of the Selaginellaceae family are mostly terrestrial, herbaceous and perennial plants and vary greatly in size, some small species have stems approximately 3 cm long, while larger species have stems 50 cm to approximately 1 m long, but under 2 cm tall. Although the Selaginellaceae family has a nearly worldwide distribution, it is not economically significant [2].

Several species of *Selaginella* are used in traditional medicine in various countries in the treatment of a variety of diseases such as skin diseases [3], gastritis [4], urinary tract infections [5], diabetes [6], hepatitis [7], cancer and cardiovascular problems [8]. Extracts from some species of *Selaginella* have demonstrated anti-inflammatory [9], anti-spasmodic [10], cytotoxic, imunnostimulant, RNA reverse transcriptase inhibitory agents [11], anti-mutagenic [12], anti-metastatic activity [13] and anti-hyperglycemic activities [14].

Previous studies involving some species of *Selaginella* revealed that this genus is a rich source of steroids [15], biflavonoids [16], alkaloids [17], secolignans [18], neolignans and caffeoyl derivatives [19]. Other compounds, such as alkaloidal glycosides, phenylpropanones and lignans, were also reported in some *Selaginella* species [20,21].

*Selaginella convoluta* is a medicinal plant found in northeastern Brazil commonly known as “jericó”, “mão-de-sapo” and “mão-fechada”. *S. convoluta* is used in folk medicine as an antidepressant [22,23], aphrodisiac, diuretic, in the treatment of amenorrhea [24], coughing, bleeding, increases female fertility [25] as well as analgesic and anti-inflammatory [26].

In our continuing search of the Brazilian Caatinga for medicinal plants to combine biodiversity conservation with drug discovery, we have previously demonstrated the antinociceptive effects of the ethanolic extract of *Amburana cearensis* in mice [27], and anti-ulcer activity of ethanolic extract of *Encholirium spectabile* in rodents [28].

To date, there are no reports on the antinociceptive activity of *S. convoluta*. Given the extensive use of this plant in the semi-arid region of Brazil to treat pain and inflammation, the aim of this study was to evaluate the antinociceptive activity of the ethanolic extract from *S. convoluta* in experimental models of pain in mice.

## Methods

### Plant material

A sample of *S. convoluta* was identified by the botanical specialist André Paviotti Fontana. The entire plant was collected in July 2009 near the city of Petrolina, state of Pernambuco, Northeastern Brazil. A voucher specimen (6440) is deposited at the Herbarium Vale do São Francisco (HVASF) of the Centro de Referência para Recuperação de Áreas Degradadas (CRAD-UNIVASF).

### Preparation of plant extract

Plant material was dried in an oven at 45°C with air circulation for three days. The dried and powdered plant (1730 g) was subjected to extraction with 95% EtOH three times at room temperature. The time of each extraction was 72 h. The extractive solution was concentrated under vacuum in a rotavapor to yield 65 g of the crude extract.

### Preliminary phytochemical screening

Preliminary phytochemical analysis of the extract was performed. The presence of alkaloids was determined with Dragendorff’s and Mayer’s reagents, flavonoids were detected with HCl and Mg powder, phenols were measured with ferric chloride and both steroids and terpenoids were detected by Liebermann-Burchard reaction [29].

### HPLC-DAD analysis of phenolic compounds

The analysis of the phenolic compound profile of the extract was performed with liquid chromatography on a Hitachi model Lachrom Elite HPLC system. The column was a LiCospher 100 RP18 (5 mm) with dimensions (150 mm × 04 mm) Merck equipped with a Diode Array Detector (DAD). The mobile phase consisted of solutions of H_2_O/H_3_PO_4_ 0.1% (A) and MeOH (B), and 75% of A and 25% of B was initially run for 25 minutes. The column temperature was maintained at 30°C with a flow rate of 1.0 ml/min. An injection volume of 20 μl of the extract was used. Spectral data were recorded in 320 nm throughout the entire procedure.

### Animals

Adult male albino Swiss mice (35–40 g) were used throughout this study. The animals were randomly housed in appropriate cages at 22 ± 2°C on a 12 h light/ dark cycle with free access to food and water. When necessary, animals were deprived of food 12 h prior to the experiments. Mice were used in groups of six or ten animals each, according to the requirements of individual experiments. The same visual observer performed all nociception tests, and efforts were made to minimise the number of animals used, as well as any discomfort to the animals. Experimental protocols and procedures were approved by the Universidade Federal do Vale do São Francisco Animal Care and Use Committee by number 024240408.

### Pharmacological tests

#### Acute toxicity

To determine the acute toxicity of the extract, the crude ethanolic extract of *S. convoluta* (Sc-EtOH) was administered intraperitoneally (2.0 g/kg) and 5.0 g/kg orally to groups of male (n = 10). The control group received vehicle. Mortality within 72 h was recorded for each group, and the animals then were observed for 7 days for signs of toxicity (body weight variation, consumption of food and water, piloerection, palpebral ptosis, locomotion and muscular tone alterations, trembling, paw paralysis, sedation, ambulation reduction, response to touch and defecation).

#### Acetic acid-induced writhing test

This test was performed using the method described by Koster et al. [30]. Mice were divided into six groups of six mice each. Acetic acid (0.9% v/v) was administered i.p. in a volume of 0.1 ml/10 g. Vehicle (saline), morphine (10 mg/kg), acetylsalicylic acid (ASA 150 mg/kg) and Sc-EtOH (100, 200 and 400 mg/kg), were administered i.p. 30 min before the injection of acetic acid. The number of abdominal constrictions produced in each group for 10 min post-injection was counted and compared to the response in the control group. Antinociceptive activity was calculated as the percentage inhibition of abdominal constriction.

#### Formalin test

The method used was similar to that described by Hunskaar and Hole [31] and Vianna et al. [32]. Twenty microliters of 2.5% formalin (in 0.9% saline, subplantar) was injected subcutaneously into the right hind paw of the mice. The time (in seconds) spent licking and biting the injected paw was measured as an indicator of pain response. Responses were measured for 5 min after formalin injection (first phase, neurogenic) and 15–30 min after formalin injection (second phase, inflammatory). Vehicle (saline), morphine (10 mg/kg), acetylsalicylic acid (ASA 150 mg/kg) and Sc-EtOH (100, 200 and 400 mg/kg) were administered i.p. 60 min before the injection of formalin. Mice were observed in the chambers with a mirror mounted on three sides to allow view of all of the paws. Antinociceptive activity was calculated as the percentage inhibition of licking time.

#### Hot plate test

Mice were divided into five groups of six mice each. Mice were pre-selected on the hot plate at 55 ± 0.5°C. Licks on the rear paws were the parameters of observation. Animals showing a reaction time (defined as the latency for licking the hind feet or jumping) greater than 20 s were discarded. The animals were then treated with vehicle (saline, 0.1 ml/10 g, i.p.), morphine (10 mg/kg, i.p.) and Sc-EtOH (100, 200 and 400 mg/kg, i.p.). Latency time (in seconds) for each mouse was determined on the hot plate during a maximum period of 20 s, at intervals of 30, 60, 90 and 120 min after the administration of the vehicle, extract and morphine [33].

#### Motor coordination test (Rota-rod test)

A rota-rod tread mill device (Insight, Brazil) was used for the evaluation of motor coordination [34]. Initially, 24 h before the test, mice capable of remaining on the rota-rod apparatus longer than 180 s (7 rpm) were selected. Thirty minutes after the administration of either Sc-EtOH (100, 200 and 400 mg/kg, i.p.), vehicle (saline/Tween 80 0.2%; control group) or diazepam (DZP; 2.5 mg/kg, i.p.), each animal was tested on the rota-rod apparatus at 0.5, 1 and 2 h post-treatment, and the time (s) the mice were able to remain on top of the bar was recorded for up to 180 s.

#### Statistical analysis

The data are expressed as the means ± S.E.M., and statistical significance was determined using an analysis of variance (ANOVA) followed by Dunnett’s test. Values were considered significant at *p* < 0.05. All analysis was performed with the GraphPad Prism 4.0 program (GraphPad Prism Software, Inc., SanDiego, CA, USA).

## Results

### Preliminary phytochemical screening

Preliminary analysis demonstrated that Sc-EtOH was positive for the presence of phenols, steroids, terpenoids and flavonoids. However, the ethanolic extract was negative for the presence of alkaloids.

### HPLC-DAD analysis of phenolic compounds

**Figure 1:**
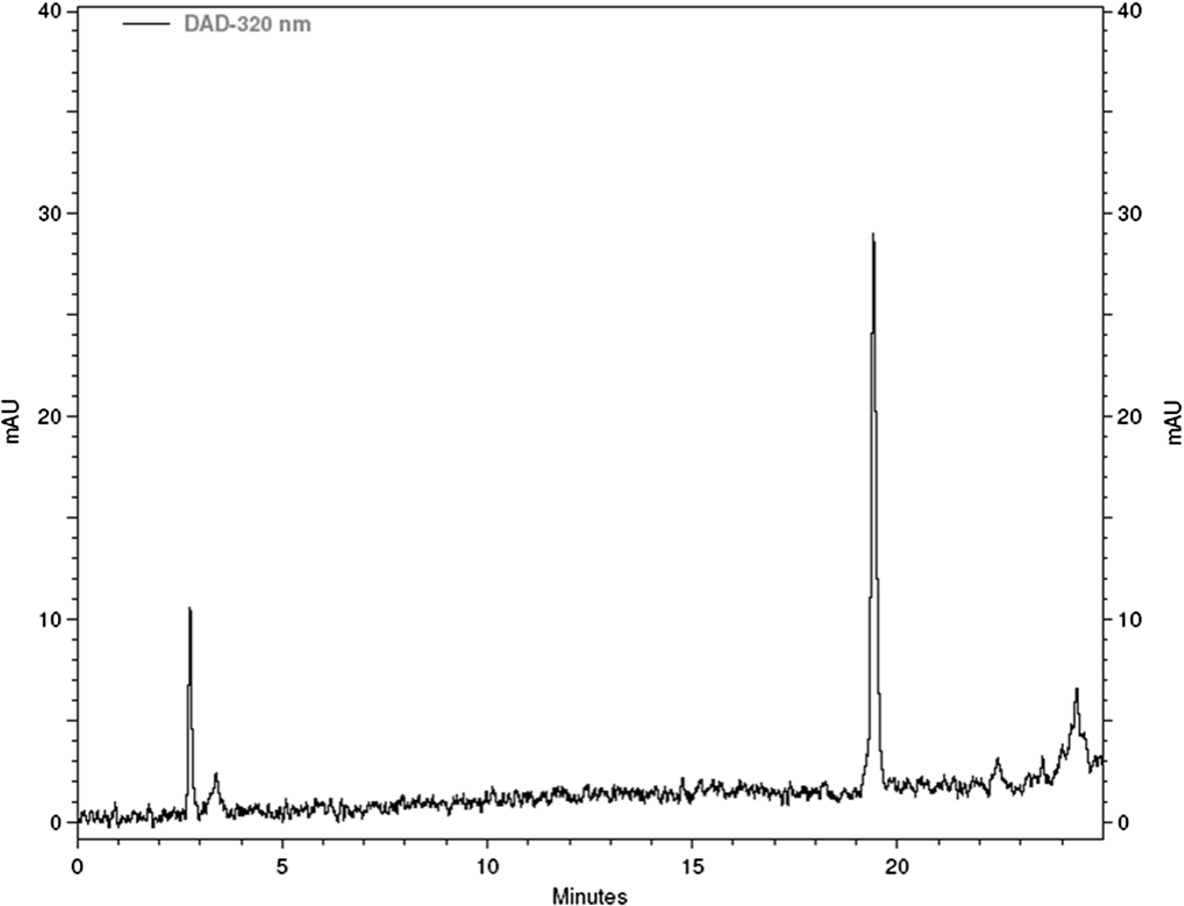
HPLC-DAD phenolic profile for the Sc-EtOH recorded at 320 nm.

The phenolic profile for Sc-EtOH at 320 nm is presented in Figure 1. The chromatogram shows the presence of a single peak with a retention time of 19.41 minutes. The compound has a UV band characteristic of flavonoids. This compound is under investigation, but in the literature review, it is reported that the plant biflavonoid amentoflavone is the major chemical constituent in different species of the genus *Selaginella*[35].

### Acute toxicity

During the evaluation for acute toxicity of Sc-EtOH, neither 2.0 g/kg intraperitoneally injected or 5.0 g/kg of orally administered Sc-EtOH resulted in death or any behavioral and/or physiological alterations, indicating that the extract has low toxicity. Further studies will be performed to confirm the absence of acute and chronic toxicity through histopathological analysis and determination of the haematological and biochemical parameters of blood.

### Acetic acid-induced writhing test

**Figure 2:**
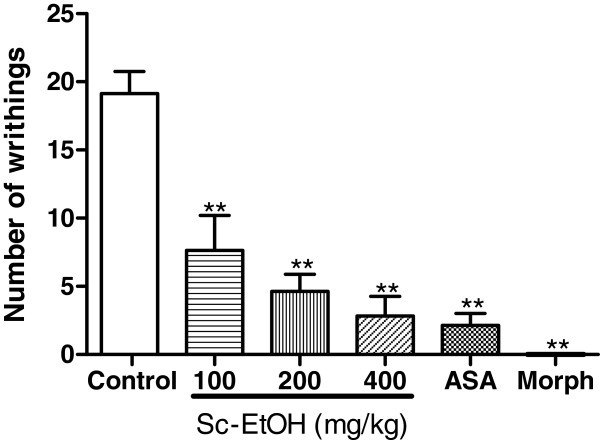
**Effect of ethanolic extract of the *Selaginella convoluta *(Sc-EtOH), acetylsalicylic acid (ASA 150 mg/kg) and morphine (Morph 10 mg/kg) on acetic acid induced writhing test.** Values are mean ± S.E.M. **p < 0.01, significantly different from control; ANOVA followed Dunnett’s test (n = 6, per group).

The intraperitoneal administration of Sc-EtOH (100, 200 and 400 mg/kg) had a dose-dependent antinociceptive effect and significantly decreased the number of writhing movements induced by the i.p. administration of the acetic acid compared with the control group (p < 0.01). The percentages of inhibition were 58.46, 75.63 and 82.23% for Sc-EtOH doses of 100, 200 and 400 mg/kg, respectively. Acetylsalicylic acid caused an 88.68% reduction in writhing movements, while morphine abolished all of the nociceptive responses (Figure 2).

### Formalin test

**Figure 3:**
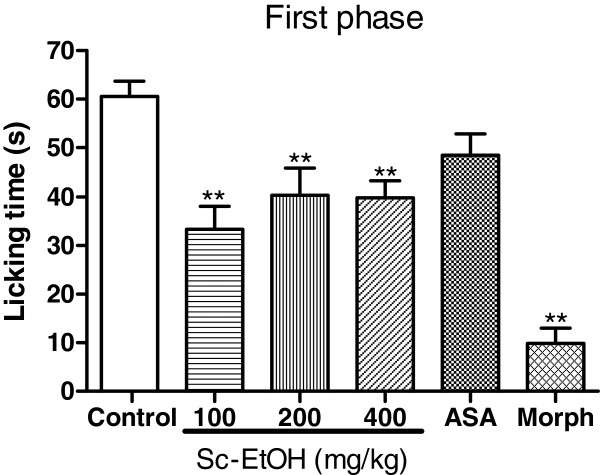
**Effect of ethanolic extract of *Selaginella convoluta *(Sc-EtOH), acetylsalicylic acid (ASA 150 mg/kg) and morphine (Morph 10 mg/kg) on formalin test (first phase).** Values are mean ± S.E.M.; **p < 0.01, significantly different from control; ANOVA followed Dunnett’s test (n = 6, per group)

**Figure 4:**
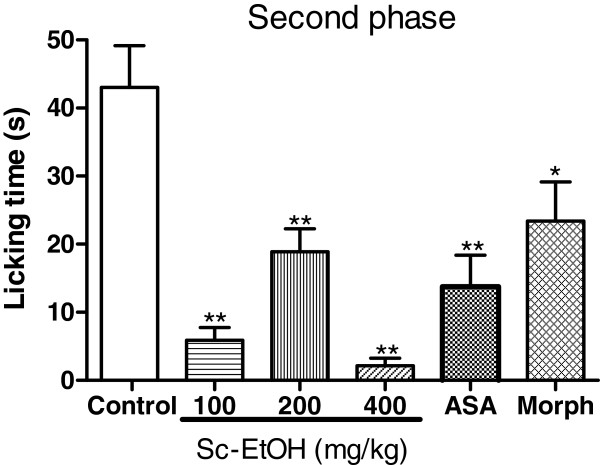
**Effect of ethanolic extract of *Selaginella convoluta *(Sc-EtOH), acetylsalicylic acid (ASA 150 mg/kg) and morphine (Morph 10 mg/kg) on formalin test (second phase).** Values are mean ± S.E.M.; *p < 0.05, **p < 0.01, significantly different from control; ANOVA followed Dunnett’s test (n = 6, per group).

The results of the formalin test are shown in Figures 3 and 4. Sc-EtOH had a significant antinociceptive effect in decreasing the paw licking time in both the neurogenic and inflammatory phases of the test, but the reduction was most significant in the second phase. Sc-EtOH administration at 100, 200 and 400 mg/kg, i.p. decreased the paw licking time by 44.90, 33.33 and 34.16%, respectively, in the first phase and by 86.44, 56.20 and 94.95%, respectively, in the second phase of the formalin test. The reference drug, acetylsalicylic acid, was effective only in the second phase (67.83%). Morphine decreased the licking time during both phases.

### Hot plate test

**Figure 5:**
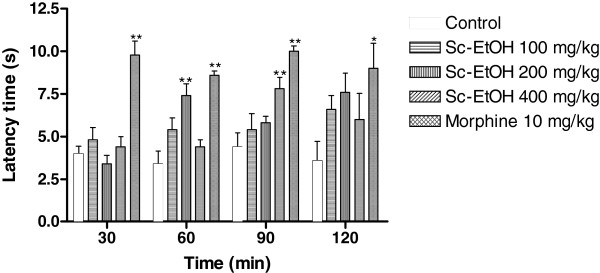
**Effect of ethanolic extract of *Selaginella convoluta *(Sc-EtOH) and morphine on hot plate test.** Values are mean ± S.E.M.; *p < 0.05, **p < 0.01, significantly different from control; ANOVA followed Dunnett’s test (n = 6, per group).

Figure 5 shows the results of the hot plate test. Sc-EtOH increased the latency time at doses of 200 and 400 mg/kg after 60 and 90 minutes, respectively. The effect of morphine (10 mg/kg) was significantly higher.

### Motor coordination test (Rota-rod test)

**Figure 6:**
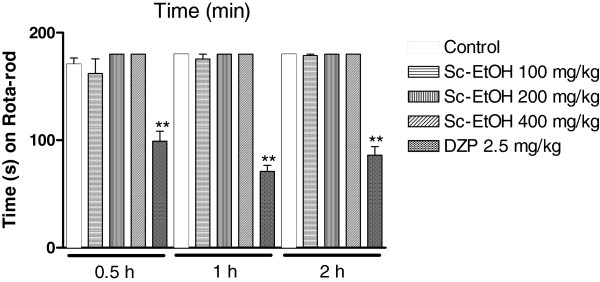
**Effect of ethanolic extract of *Selaginella convoluta *(Sc-EtOH) and diazepam (DZP) on rota-rod test.** Values are mean ± S.E.M.; **p < 0.01, significantly different from control; ANOVA followed Dunnett’s test (n = 6, per group).

In this test, Sc-EtOH did not impair motor coordination at 0.5, 1 and 2 h post-administration. Diazepam (2.5 mg/kg) caused a significant decrease in time that the animals remained on the rota-rod apparatus, compared to the control group (Figure 6).

## Discussion

The present study is the first time to demonstrate the antinociceptive activity of *S. convoluta* in classical pharmacological models of pain. Although *S. convoluta* is widely used in the folk medicine in the semi-arid region of Brazil, no report about the antinociceptive activity of this plant is recorded in the literature. The study of plant species that traditionally have been used for the relief of the pain should still be seen as a logical research strategy in the search for new analgesic drugs [36].

The first test to evaluate the antinociceptive activity of Sc-EtOH was the acetic acid-induced writhing test. The intraperitoneal administration of agents that irritate serous membranes provokes a stereotyped behaviour in the mice which is characterised by abdominal contractions, movements of the body as a whole and twisting of the dorso-abdominal muscles. This method has the advantage of detecting effects produced by weak analgesics. However, the writhing test is a non-specific method for evaluation of pain [37]. In this model pain is generated indirectly via endogenous mediators suck as bradykinin, serotonin and capsaicin, which stimulate peripheral nociceptive neurons. The release of arachidonic acid metabolites via cyclooxygenase and prostaglandin biosynthesis are also involved. Sc-EtOH administration significantly reduced acetic acid-induced writhing in mice. This result supports the hypothesis that the extract from *S. convoluta* may act by inhibiting prostaglandin synthesis because the nociceptive mechanism of abdominal writhing induced by acetic acid involves the release of arachidonic acid metabolites via cyclooxygenase (COX), and prostaglandin biosynthesis [38]. Additionally, different flavonoids have been found to be antinociceptive and anti-inflammatory agents due to their ability to inhibit arachidonic acid metabolism [39-41]. Therefore, it is possible that the presence of flavonoids in the extract of *S. convoluta* may be responsible for the antinociceptive effect. A positive result with this test is indicative of antinociceptive activity in the Sc-EtOH extract, although it remained to be determined whether this activity is of central or peripheral origin.

To distinguish between the central and peripheral antinociceptive action of Sc-EtOH, the formalin test was performed. This test is believed to represent a significant model of clinical pain [42] that involves two distinct phases. The initial phase, neurogenic pain, occurs approximately 3 min after the injection. Then, after a quiescent period, a second phase, inflammatory pain, occurs between 20 and 30 minutes post-injection [43]. The first phase results from the direct stimulation of nociceptors, whereas the second phase involves a period of sensitisation during which inflammatory phenomena occur [37]. In this experiment, Sc-EtOH decreased the licking time in both phases, but the effect was more significant in the second phase. A decrease in licking time in both phases is characteristic of drugs that act centrally and indicates a possible interaction with opioid receptors. Opioid analgesics seem to be antinociceptive for both phases, although the first phase is more sensitive to these substances. In contrast, NSAIDs, such as indomethacin and acetylsalicylic acid, seem to suppress only the second phase [31]. In the formalin test, peripheral inflammatory processes are involved in the second phase. The effect of the extract in this phase indicates that the extract has a possible anti-inflammatory effect.

The evaluation of Sc-EtOH administration with the hot plate showed that the extract presented effect in the hot plate test. The extract increased the latency time at doses of 200 and 400 mg/kg after 60 and 90 minutes, respectively. As the hot plate test is a specific central antinociceptive test, it is possible that Sc-EtOH exerts an antinociceptive effect at least in part through central mechanisms. This result is similar to what was observed in the formalin test with Sc-EtOH inhibiting of both phases of nociception.

Finally, to assess whether Sc-EtOH produces a loss of motor coordination in animals, a rota-rod test was performed. The result revealed that the extract did not produce changes in motor coordination of treated animals.

## Conclusion

It can be concluded that Sc-EtOH is effective as an analgesic agent in various pain models. The antinociceptive effect of Sc-EtOH is most likely mediated via inhibition of peripheral mediators and central inhibitory mechanisms. Our results support that *Selaginella convoluta* has therapeutic potential for the treatment of painful disorders. Further studies that are currently in progress will enable us to understand the precise mechanisms of action of Sc-EtOH.

## Competing interests

The authors declare that they have no competing interests.

## Authors’ contributions

JASF and APF were responsible for the collection and botanical identification of the specie. XPN and PGSS were responsible for preparation of the extract. PKFD, CRCB and AB were responsible for phytochemical studies and HPLC-DAD analysis of the extract. JSS, LJQJ and JRGSA conducted the acute toxicity and antinociceptive activity assays, analyzed and interpreted the data, and drafted the manuscript. JTL was responsible for the rota-rod test. All authors read and approved the final manuscript.

## Pre-publication history

The pre-publication history for this paper can be accessed here:

http://www.biomedcentral.com/1472-6882/12/187/prepub
